# Synthesis of a Rationally Designed Multi-Component Photocatalyst Pt:SiO_2_:TiO_2_(P25) with Improved Activity for Dye Degradation by Atomic Layer Deposition

**DOI:** 10.3390/nano10081496

**Published:** 2020-07-30

**Authors:** Dominik Benz, Hao Van Bui, Hubertus T. Hintzen, Michiel T. Kreutzer, J. Ruud van Ommen

**Affiliations:** 1 Group Product & Process Engineering, Department of Chemical Engineering, Faculty of Applied Sciences, Delft University of Technology, 2629 HZ Delft, The Netherlands; 2 Faculty of Electrical and Electronic Engineering, Phenikaa University, Yen Nghia, Ha-Dong District, Hanoi 12116, Vietnam; hao.buivan@phenikaa-uni.edu.vn; 3 Phenikaa Research and Technology Institute (PRATI), A&A Green Phoenix Group, 167 Hoang Ngan, Hanoi 10000, Vietnam; 4 Group Luminescent Materials, Section Fundamental Aspects of Materials and Energy, Faculty of Applied Sciences, Delft University of Technology, 2629 HZ Delft, The Netherlands; h.t.hintzen@tudelft.nl; 5 Faculty of Science, Leiden University, 2333 CC Leiden, The Netherlands; m.t.kreutzer@science.leidenuniv.nl

**Keywords:** atomic layer deposition, photocatalysis, dye degradation, TiO_2_, multicomponent material

## Abstract

Photocatalysts for water purification typically lack efficiency for practical applications. Here we present a multi-component (Pt:SiO_2_:TiO_2_(P25)) material that was designed using knowledge of reaction mechanisms of mono-modified catalysts (SiO_2_:TiO_2_, and Pt:TiO_2_) combined with the potential of atomic layer deposition (ALD). The deposition of ultrathin SiO_2_ layers on TiO_2_ nanoparticles, applying ALD in a fluidized bed reactor, demonstrated in earlier studies their beneficial effects for the photocatalytic degradation of organic pollutants due to more acidic surface Si–OH groups which benefit the generation of hydroxyl radicals. Furthermore, our investigation on the role of Pt on TiO_2_(P25), as an improved photocatalyst, demonstrated that suppression of charge recombination by oxygen adsorbed on the Pt particles, reacting with the separated electrons to superoxide radicals, acts as an important factor for the catalytic improvement. Combining both materials into the resulting Pt:SiO_2_:TiO_2_(P25) nanopowder exceeded the dye degradation performance of both the individual SiO_2_:TiO_2_(P25) (1.5 fold) and Pt:TiO_2_(P25) (4-fold) catalysts by 6-fold as compared to TiO_2_(P25). This approach thus shows that by understanding the individual materials’ behavior and using ALD as an appropriate deposition technique enabling control on the nano-scale, new materials can be designed and developed, further improving the photocatalytic activity. Our research demonstrates that ALD is an attractive technology to synthesize multicomponent catalysts in a precise and scalable way.

## 1. Introduction

Titania (TiO_2_) was first discovered as an active photocatalyst almost half a century ago [1] and still serves as a widely used benchmark for the development of new photocatalytic materials or as a substrate material to improve their photocatalytic properties [2,3,4]. The photocatalytic performance combined with affordable price, stability, and non-toxicity are a few reasons for this success in scientific publications. However, its implementation in real applications, despite these advantages, is hampered by poor sunlight utilization, due to a large bandgap, and charge recombination resulting in insufficient efficiency [5,6,7]. Improving TiO_2_ by incorporating several elements into the lattice (bulk modification) or depositing different materials onto the surface (surface modification) has been extensively explored ever since [8,9,10,11,12,13]. This includes modifications with both noble metals and metal oxide for the improvement in: (1) reactant adsorption and reactivity by surface modification, (2) light absorption by bandgap modification, (3) charge utilization by modification of the band levels. These three approaches are dominantly targeted to develop a better catalyst. On the other hand, especially improvements in the rates and mechanisms of radical generation are often underestimated since they are often attributed to the improved light absorption or reduced charge recombination. However, it has been shown that surface modifications, which lead to a different type and amount of terminal OH groups, result in an increased OH radical generation [14]. Factors such as adsorption of the reactants for the ROS formation have been demonstrated to play a crucial role in the enhancement of the photocatalytic activity [15,16,17]. Overall, the photocatalytic mechanism gives four different points to tackle for making improved materials [18,19]:

(1) Adsorption of reactants.
(2) Creation of charge carriers by light absorption.
(3) Charge carrier separation.
(4) Degradation reactions, along:
  (a) Conduction band pathway via superoxide radicals (converting into hydroxyl radicals).
  (b) Valence band pathway via direct oxidation.
  (c) Valence band pathway via hydroxyl radicals.

While earlier material development focused more on the enhancement using single modifications on active materials, recently, multicomponent systems have received more attention [20,21,22,23,24,25]. However, most works on multicomponent materials focus on combining materials where components improve the processability of the catalyst for their implementation in future reactors (i.e., dispersion, separation using magnets [21,26]). Multicomponent materials containing iron oxide as a core material, due to its magnetic properties, are good examples of materials improving both the processability and activity of a catalyst to enhance separation from the liquid after the reaction [21,24]. Another approach is to modify the surface of an already photocatalytic material with a coating to change the deposition properties of the subsequently added co-catalyst i.e., increasing control of the particle size by modifying the surface or preventing the agglomeration of deposited nanoclusters of the surface by a cover layer [27,28,29,30]. However, the manipulation of physical properties such as increased surface area, the addition of magnetic properties, or a beneficial layer to improve the dispersion of co-catalyst particles [31,32] is more straightforward than modifying the photocatalytic mechanisms themselves. The question arises whether multiple components, each enhancing the photocatalytic process, can be combined to reach higher enhancement than each component individually.

The approach of combining materials to make a more complex and improved structure can be tackled utilizing the advantages of atomic layer deposition. This technology allows to precisely control the deposition of various materials onto a surface. The advantages of confined scalable bottom-up building up of multicomponent catalyst materials could lead to the development of improved photocatalysts. However, building up multicomponent catalysts requires detailed knowledge of the photocatalytic working principles of the base materials as well as the building blocks and their interaction.

Surface deposited Pt on TiO_2_(P25) is a well-developed catalyst and has shown superior activity for the photocatalytic degradation of various organic pollutants [33,34]. Pt enhances the photocatalytic activity of TiO_2_(P25) for dye degradation by acting as an adsorption surface for O_2_ suppressing charge carrier recombination by facilitating improved radical generation via the conduction pathway in the presence of dissolved O_2_ [15,35]. Our previous findings for the role of an ultrathin SiO_2_ coating on TiO_2_(P25) nanoparticles demonstrated an improved photocatalytic activity due to the improved generation of OH radicals at the SiO_2_ surface from the more efficiently separated holes on the SiO_2_ surface by the more acidic Si-OH surface groups [14,16], which is different from TiO_2_(P25) as well as Pt:TiO_2_(P25). From this detailed analysis of the photocatalytic mechanisms, the question arises whether the advantages of these bi-material composites, Pt:TiO_2_(P25) and SiO_2_:TiO_2_(P25), can be combined in a tri-material composite resulting in a further improved photocatalyst for dye degradation. Here, we demonstrate that catalyst development following the design principles of combining characteristics that are improving individual aspects of the photocatalytic reaction pathways can lead to superior photocatalysts by taking advantage of the controllability of the deposition using ALD.

## 2. Experimental Section

### 2.1. Synthesis

TiO_2_ nanoparticles (P25, mean diameter ~21 nm, specific surface area of ~54 m^2^ g^−1^ measured by BET) was purchased from Evonik Industries (Hanau, Germany). Silicon tetrachloride (SiCl_4_) and trimethyl(methylcyclopentadienyl)platinum(IV) (MeCpPtMe_3_) were purchased from AlfaAesar (Karlsruhe, Germany) and Strem Chemicals (Bischheim, France), respectively. Both chemicals were stored in a stainless steel bubbler for mounting into the ALD setup. Acid Blue 9 (Brilliant Blue FCF) and sodium polyphosphate were purchased from SigmaAldrich (Zwijndrecht, The Netherlands) and used without further purification. Pt:SiO_2_:TiO_2_(P25) was synthesized via a two-step deposition process:

(1) SiO_2_ was deposited on TiO_2_(P25) on a homebuilt ALD setup in a fluidized bed under atmospheric pressure, as described in detail elsewhere [36,37] In brief, 5 g of the TiO_2_(P25) powder was put in a quartz glass column (diameter 26 mm, height 500 mm), which was then placed on a vertical vibration table (Paja 40/40-24, Oosterhout, The Netherlands) to assist fluidization. The powder was sieved prior to the ALD experiments with a mesh size of 250 µm to break or exclude larger agglomerates. SiO_2_ layers were deposited using SiCl_4_ and H_2_O as precursors, which were both kept at room temperature in stainless steel bubblers. The reactor was heated to 100 °C throughout the deposition process using an IR lamp. For different SiO_2_ loadings, up to 40 cycles were applied using an exposure time of 30 s SiCl_4_ and a 3 min H_2_O pulse. Purging steps of nitrogen for 3 min and 8 min, respectively, separated the precursor pulses.
(2) The SiO_2_:TiO_2_(P25)powder was split into 1.5 g batches, which were then used for the deposition of Pt clusters on the SiO_2_:TiO_2_(P25)surface using MeCpPtMe_3_and O_2_ as precursors. The Pt precursor was stored in stainless steel bubblers and held at 70 °C. For those experiments, one ALD cycle was performed using exposure times for the Pt precursor ranging from 20 sec to 5 min. The O_2_ exposure was set to 5 min, and both precursor exposures were separated using purge steps of 5 min with Nitrogen, respectively. Afterward the coated powders were treated under an atmosphere of 5% H_2_ in N_2_ (*v*/*v*) in a fixed bed reactor. The temperature was ramped up from room temperature to 200 °C with a rate of 2 °C/min and then was held constant for 5 min after which the powder was allowed to cool to room temperature.

### 2.2. Characterization

For the ICP-OES analysis, approximately 30 mg of sample was digested in a mixture of 4.5 mL 30% HCl, 1.5 mL 65% HNO_3_, and 0.2 mL 40% HF using a microwave for 60 min. After the digestion, the samples were diluted to 50 mL with MilliQ water, and the weight percentage (wt. %) of Pt, Si, and Ti were analyzed with a Perkin Elmer ICP-OES 5300DV (Waltham, MA, USA) system. The samples were also diluted 20 times for the analysis of the Ti concentration. TEM micrographs were acquired from a JEOL JEM1400 transmission electron microscope (Akishima, Japan) at 120 kV. As-deposited Pt:SiO_2_:TiO_2_(P25) nanoparticles were suspended in ethanol and transferred to Cu transmission electron microscopy grids (3.05 mm in diameter, Quantifoil, Großlöbichau, Germany). X-ray photoelectron spectra (XPS) were recorded on a ThermoFisher K-Alpha (Blijswik, The Netherlands) system using Al Kα radiation with a photon energy of 1486.7 eV. A sufficient amount of powders was immobilized on copper tape before loading into the XPS chamber. Survey scans were acquired using a 400 μm spot size, 55 eV pass energy, and 0.1 eV/step with charge neutralization. The peak positions were calibrated by referencing the C 1s peak to 284.8 eV and previous background subtraction using the Thermo Avantage software (v5.985, accessed on 19 February 2019).

### 2.3. Photocatalytic Testing

The photocatalytic activity was evaluated in a 100 mL glass bottle (irradiation surface 11.3 cm^2^) with a 30 mL solution of Acid Blue 9 (16 mg/L in deionized water) and 30 mg of catalyst powder. Sodium polyphosphate (0.3 mL, 100 g/L in water) was added to aid the dispersion of the powder [38]. The powder was dispersed by sonicating the solution with the powder for 10 min. Additionally, the dispersion was stirred in the dark for another 20 min in order to reach the dye-adsorption-desorption equilibrium. The photocatalytic test was executed in open air in an Atlas SunTest XXL (Rycobel, Deerlijk, The Netherlands) solar simulator equipped with three xenon lamps to ensure homogeneous light distribution (top illumination). The irradiance was set to 45 W/m^2^ at the reactor surface with the reactor placed about 40 cm distant from the lamps. In order to ensure a constant temperature during the experiment the reactor was placed in a water bath maintaining a temperature of 20 °C. Multiple samples were irradiated on a multiple stirring plate (700 rpm). Samples of 1 mL were taken after distinct times of irradiation, were subsequently centrifuged, and the dye concentration in the supernatant liquid was determined using a UV/Vis spectrometer (DR5000 Hach-Lange, Düsseldorf, Germany). The absorption was measured at 629 nm, which represents the maximum absorption for Acid Blue 9. Assuming 1st order kinetics, ln(C_0_/C_t_) was plotted vs. time, and the slope of the linear regression represents the kinetic constant.

## 3. Results and Discussion

**Figure 1:**
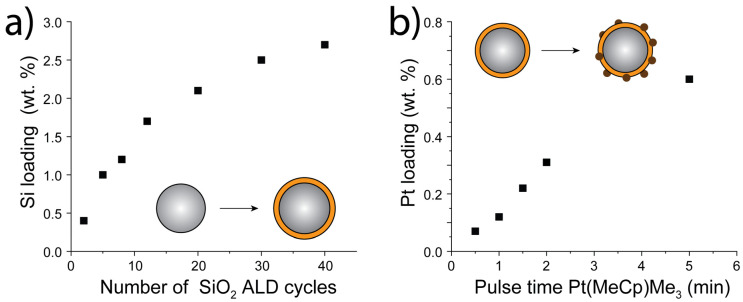
(**a**) Development of the Si loading with the number of applied SiO_2_ ALD cycles on TiO_2_(P25) particles, (**b**) Development of the Pt loading with the Pt precursor pulse time (MeCpPtMe_3_) on SiO_2_ coated TiO_2_(P25) particles (2.1 ± 0.2 wt. % Si).

Previously, we have reported on the mechanisms of SiO_2_:TiO_2_(P25) [16] and Pt:TiO_2_(P25) [35] for the photocatalytic degradation of organic pollutants. With an optimum loading of 1.7 wt. % Si we concluded that for TiO_2_(P25), a very thin SiO_2_ layer combined improve charge separation with an increased OH radical formation due to more acidic surface OH groups at the SiO_2_ surface leading to a higher degradation rate of organic pollutants in water. Too thick an SiO_2_ layer shields off the TiO_2_ and drastically reduces the photocatalytic activity. For Pt:TiO_2_(P25), the role of dissolved O_2_ in combination with the enhanced charge separation turned out to be crucial for the enhancement of the photocatalytic activity. Pt acts as a recombination center and reduces the photocatalytic activity if the electrons cannot be harvested adequately in case no O_2_ is present or the Pt loading is too high. This gave rise to the improved behavior for Pt:TiO_2_(P25) samples with an optimal Pt loading of 0.34 wt. %. Higher loadings decreased the photocatalytic activity even to lower values than intrinsic TiO_2_(P25) (>2 wt % Pt). In earlier research, SiO_2_ layers often have been used to encapsulate Pt nanoclusters on the surface of TiO_2_ to improve the Pt nanoparticle dispersion by preventing noble metal cluster agglomeration, improving the catalytic performance [31,32,39]. According to our previous results, it would be more advantageous to keep Pt clusters exposed on the surface by the deposition of SiO_2_ followed by Pt, since both materials, Pt and SiO_2_, are crucial for the generation of radicals on the surface. The overcoating of Pt with SiO_2_ would reduce the effect of Pt adsorbing O_2_ and possibly even give a worse photocatalyst due to the charge recombination property of the Pt nanoclusters in case the lack of access to O_2_ prevents the harvest of electrons. Therefore, for the material design, we chose to coat first the TiO_2_(P25) nanoparticles with SiO_2_ and subsequently deposit Pt onto the SiO_2_:TiO_2_(P25)substrate (Figure 1a). Despite the spatial separation of the Pt clusters from TiO_2_, electrons may still be able to transfer to the Pt clusters as for the SiO_2_:TiO_2_(P25) catalyst excited electrons may still reach through the ultrathin SiO_2_ layer to be harvested [16]. For low Si loadings the layer may be porous and incomplete which would also give the chance for a reaction of the electrons directly on the TiO_2_ surface. By applying 2 to 40 ALD cycles of SiCl_4_ and H_2_O the loading evolved from 0.4 to 2.7 wt % Si in a conformal manner (Figure 1a). Afterward, Pt was deposited onto SiO_2_:TiO_2_(P25) in various loadings by using different pulse times (20 s to 5 min) for MeCpPtMe_3_ and O_2_ as a counter reactant (Figure 1b). In order to reach the required very low Pt loadings the pulse time was in the undersaturation regime resulting in a dependency of the pulse time versus Pt loading rather the commonly used cycle number. Using this approach, we could precisely tune the loadings for both the SiO_2_ coating and the Pt clusters.

**Figure 2:**
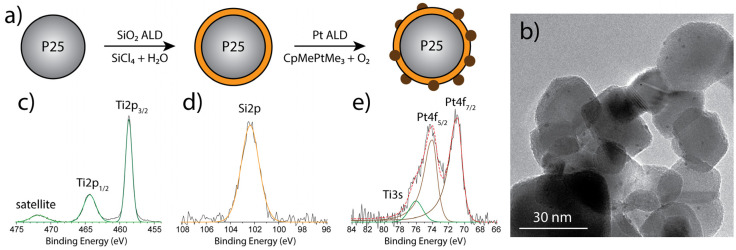
(**a**) Deposition scheme for the material development of Pt:SiO_2_:TiO_2_(P25), (**b**) TEM picture of Pt:SiO_2_:TiO_2_(P25) (1 wt. % Si, 1.23 wt. % Pt), grey large particle are P25 particles, SiO_2_ layer around P25 particles can be observed, dark spots indicate Pt nanoclusters, (**c**) Ti 2p HRXPS spectrum with typical TiO_2_ features, (**d**) Si 2p HRXPS spectrum, single peak represents SiO_2_ (orange), (**e**) Pt 4f HRXPS spectrum, doublet peak represents Pt metal (brown), single peak represents Ti 3s satellite peak (green).

This design of experiments gives a matrix of various combinations of SiO_2_ and Pt loadings onto P25 nanoparticles. Analyzing the morphology with TEM revealed the growth of SiO_2_ as conformal sub-nanometer layers layers with various loading(Figure 2a) onto P25 and the Pt clusters (1–2 nm) deposited onto the SiO_2_ layers in the second step (Figure 2b, Figure S1). This is in agreement with our previous studies that show the growth of layers of SiO_2_ due to the high affinity towards the TiO_2_ surface [17]. On the other hand, the high surface energy of Pt results in the expected island growth on the surface of SiO_2,_ resulting in similar behavior as the deposition of Pt clusters on P25 itself [40]. Due to the very minute addition of material on the P25 particles TEM pictures confirms an unchanged particle size of Pt:SiO_2_:TiO_2_(P25) particles compared to pristine P25 of about 30 nm (Figure 2b). For lower loaded SiO_2_ samples, the layers are not clearly visible due to resolution limitations of the TEM. It is expected that for a low number of cycles, the SiO_2_ layer may be incomplete, and the TiO_2_ surface is still exposed. XPS confirmed the deposition of both materials, SiO_2_ and Pt (Figure 2c–e). From the high-resolution spectra, we can identify the characteristic peaks for each element – Ti, Si, and Pt. For the range between 450 eV and 475 eV, the characteristic Ti(IV) peaks for TiO_2_ can be observed at binding energies of 458.68 eV (Ti 2p_3/2_) and 464.34 eV (Ti 2p_1/2_). The Si 2p peak characteristic for SiO_2_ arises at 102.36 eV. After deconvolution of the Pt HR-XPS spectrum, three peaks arise where the peaks at 70.90 eV and 74.01 eV can be fitted to Pt(0) 4f_7/2_ and Pt(0) 4f_5/2_ respectively correlating them to the metallic Pt species, and at a binding energy of 76.06 eV, a satellite peak from the Ti 3s peak arises. XRD studies on TiO_2_(P25) after modification with SiO_2_ and Pt using ALD showed no change in the TiO_2_ crystal structure featuring anatase and rutile phase typical for the mixed phase P25 nanoparticles (Figure S3). The modified TiO_2_(P25) showed a peak at 46.6° indicating the Pt(200) facet. However, other facets of Pt were not observable. Due to the only minute changes, i.e., ultra-thin layers of SiO_2_ on the surface of TiO_2_ XRD features of SiO_2_ remain undetected. UV/Vis absorbance spectra (measured with DRS) show that the addition of neither SiO_2_ layers nor Pt clusters leads to a significant change in the bandgap of the modified catalysts (Figure S4). The deposition of Pt clusters leads (for both TiO_2_(P25) itself as well as SiO_2_:TiO_2_(P25)) to strong absorption throughout the visible spectrum caused by the metallic particles on the surface of the catalysts. Previous studies on the effect of the BET surface area of the single modified materials SiO_2_:TiO_2_(P25) (54 m^2^/g) [17] and Pt:TiO_2_(P25) (55 m^2^/g) [35] demonstrated that a minute additions of SiO_2_ and Pt are not significantly affecting the BET surface area compared to pure TiO_2_(P25) (55 m^2^/g). Therefore we expect a similar BET surface area for the bi-modified Pt:SiO_2_:TiO_2_(P25) photocatalyst. Furthermore, the adsorption of Acid Blue 9 on the surface of the prepared catalysts after reaching the adsorption-desorption equilibrium did not change significantly upon modification of TiO_2_(P25) (Figure S5).

**Figure 3:**
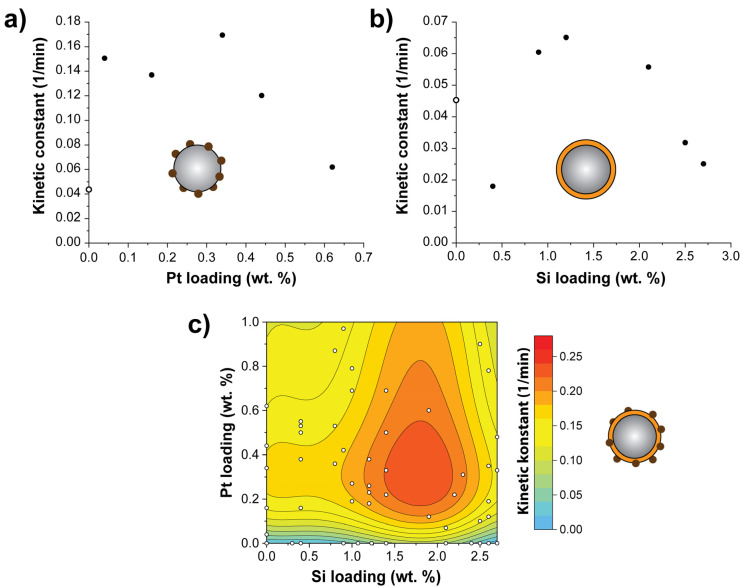
Kinetic constant for degradation of AB9 (**a**) with different loadings of Pt on TiO_2_(P25), (**b**) with different loadings of SiO_2_ on TiO_2_(P25), (**c**) combined Pt:SiO_2_:TiO_2_(P25) material with different loadings of Pt and Si (fitted results, see also Figure S2).

In order to investigate the photocatalytic performance of the combined material (Pt:SiO_2_:TiO_2_(P25)), it is compared to the original material (TiO_2_(P25)) and the single-modified materials – Pt:TiO_2_(P25) and SiO_2_:TiO_2_(P25). First, the photocatalytic degradation for those individual compounds is investigated to benchmark the measurements. As compared to TiO_2_(P25), the optimal Pt loading of about 0.34 wt. % gives a 4-fold increase in the activity of Pt:TiO_2_(P25) for the photocatalytic degradation of AB9 (Figure 3a) while SiO_2_:TiO_2_(P25) shows a 1.5-fold increased photocatalytic activity with an optimal loading of about 1.2 wt. % Si (Figure 3b). In our previous research, an optimal loading of about 1.8 wt. % Si was reported, which is in line with the results in this paper [16].

In Figure 3, we show the photocatalytic degradation of Acid Blue 9 for different coated Pt:SiO_2_:TiO_2_(P25) samples with various SiO_2_ and Pt loading in order to find the optimum loading. We can identify an optimal behavior in the range of 1.5–2.2 wt. % Si and around 0.3–0.4 wt. % Pt (Figure 3c). This range is also covering the ranges of individual catalysts SiO_2_:TiO_2_(P25)and Pt:TiO_2_(P25) perform in an optimal way. The best performing catalyst of the Pt:SiO_2_:TiO_2_(P25) series (1.9 wt. % Si, 0.60 wt. % Pt) degrades AB9 about six times better than P25. Evidently, the combination of Pt nanoparticles onto sub-nanometer layers of SiO_2_ coated on P25 particles results in an improved photocatalytic material. Although the best performing catalyst with 0.60 wt. % Pt is slightly outside the range of the optimal loading (Table 1, Figure S2) for Pt:TiO_2_(P25) mono-modified (0.34 wt. %), the fitting of the data (2D polynomial model) reveals that the optimal loading for the mono-modified materials Pt:TiO_2_(P25) and SiO_2_:TiO_2_(P25) coincides with the optimal loadings for these mono-modified catalysts. This indicates that there is limited interaction between the improvement mechanisms of depositing SiO_2_ and Pt onto P25. In this context it may be speculated that the growth of Pt on the SiO_2_ coating may positively influence particle size distribution due to a change in surface energy. However, a study on the detailed evolution of the particle size distribution of Pt clusters on different surfaces would be outside of the scope of this study yet important for further research.

On the one hand, a sub-nanometer SiO_2_ layer on TiO_2_(P25) particles improves the OH radical generation at the SiO_2_ surface from the holes generated in the VB. On the other hand, Pt clusters facilitate effective electron-hole-pair separation by using the TiO_2_ CB electrons as reducing agents to generate superoxide radicals (O_2_^−.^) from dissolved O_2_. Combining both materials, SiO_2_ and Pt, with TiO_2_(P25) increases the photocatalytic activity by increasing mainly the radical generation.

The current study does not show any serious deactivation of the photocatalyst during the use of the photocatalyst. Nevertheless, a detailed analysis of the long-term reusability will be required for the implementation in future reactors for water treatment and is recommended to be subject to follow-up research. Applying the same strategy followed in this research to improve the photocatalytic activity using a base material with increased light absorption could further increase the overall performance. For example, N-doping has been shown to reduce the bandgap of titania, increasing the light absorption in the visible light regime [41,42]. Adding another material on the surface is, on the other hand, not recommended. This would probably lead to coverage of the photocatalytic relevant surface groups reducing the reactive oxygen species generation. Furthermore, the modification of TiO_2_ by layer/nanocluster combination using cheaper materials as replacement for e.g., Pt may result in both efficient and cheaper catalysts. In our experiments we were able to coat powder of up to 5 g (~270 m^2^ surface area) using atomic layer deposition in a fluidized bed. This technique provides therefore a great opportunity to scale up the preparation of multicomponent catalysts.

## 4. Conclusions

This work shows that by the smart combination of different materials onto TiO_2_(P25), better photocatalyst can be engineered using the excellent precision of ALD in a fluidized bed. As a successful example we designed a Pt:SiO_2_:TiO_2_(P25) material with improved photocatalytic properties by depositing Pt clusters on SiO_2_ coated TiO_2_(P25). The choice of the coating materials Pt and SiO_2_ was based on our previous detailed analysis of the photocatalytic mechanism of both SiO_2_:TiO_2_(P25) and Pt:TiO_2_(P25). ALD provided the opportunity to precisely control the loading of SiO_2_ and Pt, resulting in a matrix of various loaded Pt:SiO_2_:TiO_2_(P25) surface modified nanoparticles. We showed that this material accelerates the photocatalytic degradation rate of Acid Blue 9 by a factor 6, which is superior to both SiO_2_:TiO_2_(P25) (1.5×) and Pt:TiO_2_(P25) (4×).

## Figures and Tables

**Table 1:** Kinetic constants for the best performing photocatalysts for the mono-modified and bi-modified TiO_2_ catalysts. Kinetic constants are calculated form pseudo 1st order kinetics.

	P25	Pt:TiO_2_(P25)	SiO_2_:TiO_2_(P25)	Pt:SiO_2_:TiO_2_(P25)
Optimal Loading	–	0.34 wt. % (Pt)	1.2 wt. % (Si)	0.6 wt. % (Pt) 1.9 wt. % (Si)
Kinetic Constant (min^−1^)	0.044	0.169	0.065	0.267
Improvement Ratio	1	3.84	1.48	6.07

## Supplementary Materials

The following are available online at https://www.mdpi.com/2079-4991/10/8/1496/s1, Figure S1: TEM pictures of Pt:P25 and SiO2:25, Figure S2: raw data photocatalytic activity, Figure S3: XRD spectra, Figure S4: UV/Vis DRS spectra, Figure S5: dye adsorption on catalyst surface.
