# Supplementary material for: Synthesis of a Rationally Designed Multi-Component Photocatalyst Pt:SiO2:TiO2(P25) with Improved Activity for Dye Degradation by Atomic Layer Deposition
Source: Nanomaterials (Basel). 2020 Jul 30;10(8):1496. doi: 10.3390/nano10081496 (PMC7466466; doi:10.3390/nano10081496)
Supplement: Supplementary file 1 [file nanomaterials-10-01496-s001.pdf]

nanomaterials

MDPI

Synthesis of a Rationally Designed Multi-Component Photocatalyst Pt:SiO2:TiO2(P25) with Improved Activity for Dye Degradation by Atomic Layer Deposition

Dominik Benz [1,*], Hao Van Bui [2,3], Hubertus T. Hintzen [4], Michiel T. Kreutzer [5] and J. Ruud van Ommen [1,*]

[1] Group Product & Process Engineering, Department of Chemical Engineering, Faculty of Applied Sciences, Delft University of Technology, 2629 HZ Delft, The Netherlands

[2] Faculty of Electrical and Electronic Engineering, Phenikaa University, Yen Nghia, Ha-Dong District, Hanoi 12116, Vietnam; hao.buivan@phenikaa-uni.edu.vn

[3] Phenikaa Research and Technology Institute (PRATI), A&A Green Phoenix Group, 167 Hoang Ngan, Hanoi 10000, Vietnam

[4] Group Luminescent Materials, Section Fundamental Aspects of Materials and Energy, Faculty of Applied Sciences, Delft University of Technology, 2629 HZ Delft, The Netherlands; h.t.hintzen@tudelft.nl

[5] Faculty of Science, Leiden University, 2333 CC Leiden, The Netherlands; m.t.kreutzer@science.leidenuniv.nl

* Correspondence: d.benz@tudelft.nl (D.B.); j.r.vanommen@tudelft.nl; Tel.: +31 52782133 (J.R.v.O.)

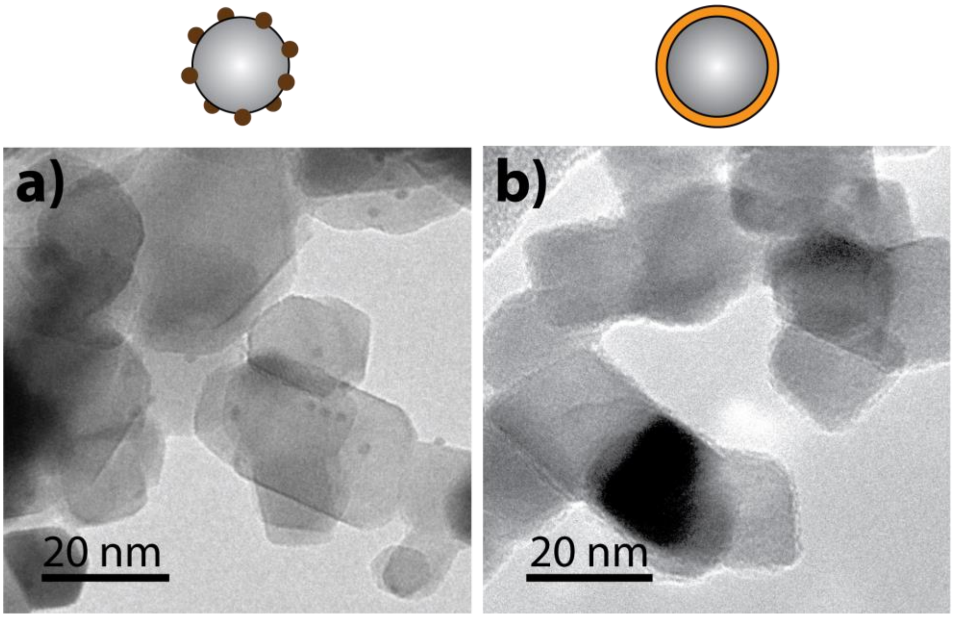

Figure S1. TEM pictures of mono modified catalysts; (**a**) Pt:TiO_2(P25) (0.34 wt % Pt), (**b**) SiO_2:TiO_2(P25) (1.7 wt % Si).

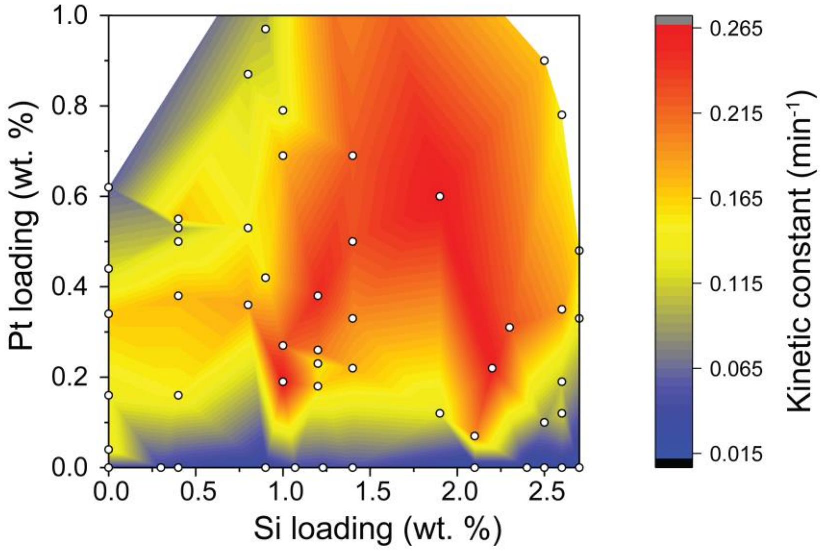

Figure S2. Contour plot for the unfitted photocatalytic activity degrading Acid Blue 9 with the multicomponent material Pt:SiO_2:TiO_2(P25).

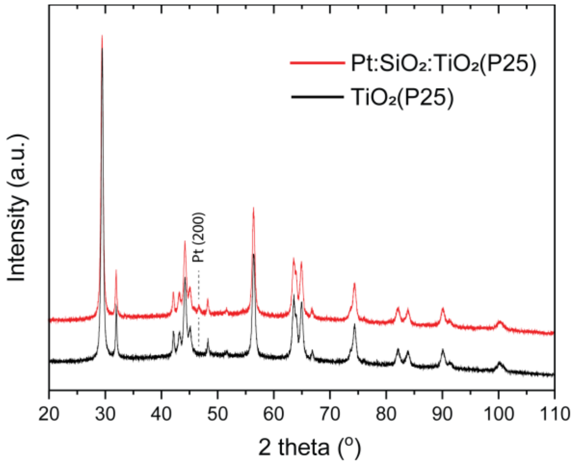

Figure S3. XRD pattern of bare TiO_2(P25) (black) and Pt:SiO_2:TiO_2(P25) (red) with the characteristic phase composition for anatase/rutile mixed phase TiO_2(P25) nanopowder.

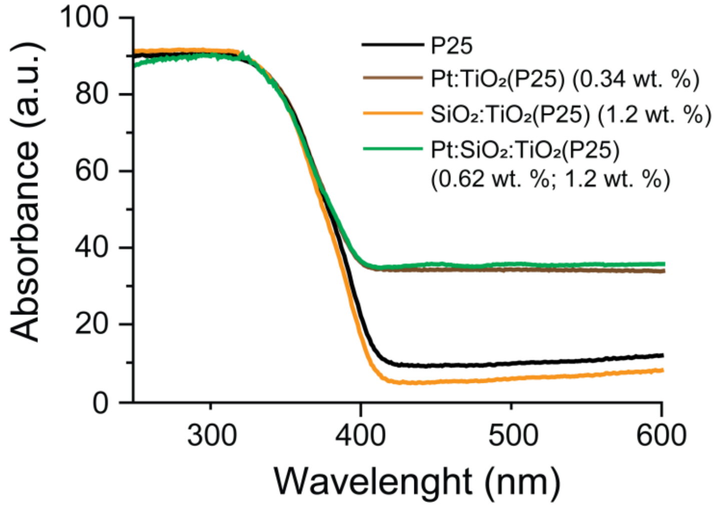

Figure S4. UV/Vis DRS spectra of the deposited catalysts with TiO_2 (P25) as a reference (black), SiO_2:TiO_2(P25) (orange), Pt: TiO_2(P25) (brown), Pt:SiO_2: TiO_2(P25) (green).

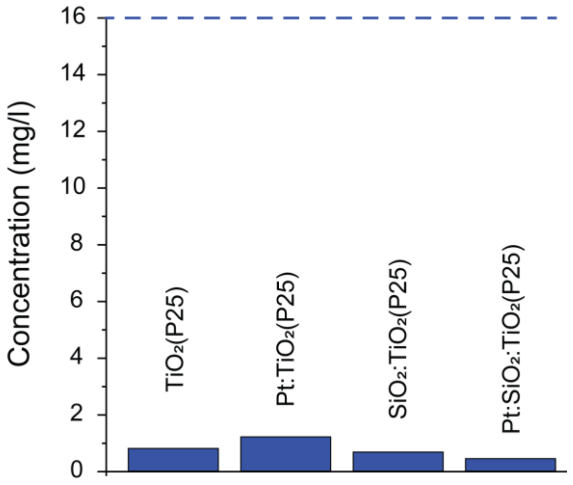

Figure S5. Adsorbed Acid Blue 9 on the catalyst surface after reaching the adsorption desorption equilibrium. The dashed line represents the original concentration of Acid Blue 9 in the solution as described in the Experimental section.
